# Assessment of Efficacy and Safety of Mangifera indica Extract (Stadice®) for Cognitive Function: A Randomized, Double-Blind, Placebo-Controlled Study

**DOI:** 10.7759/cureus.65751

**Published:** 2024-07-30

**Authors:** Shankaranarayanan Jeyakodi, Arunkanth Krishnakumar, Meena Dalal, B. Sohandas Shetty

**Affiliations:** 1 Research and Development, Zeus Hygia Lifesciences Pvt Ltd, Hyderabad, IND; 2 Clinical Research, TrialGuna Private Limited, Bengaluru, IND

**Keywords:** learning and memory, cognitive health, alertness, focus, esports, mangifera indica, mangiferin, stadice®

## Abstract

Background and objectives

Stadice^®^ is a proprietary herbal ingredient preparation standardized to mangiferin, developed to support cognitive wellness in healthy adults. *Mangifera indica* extract and its active constituent, mangiferin were known for its positive cognitive health benefits at experimental levels. This was an attempt to evaluate the clinical efficacy and safety of Stadice^®^ on healthy subjects.

Materials and methods

A randomized, double-blind, placebo-controlled clinical study was designed to study the efficacy and safety of Stadice^®^. Sixty healthy subjects who were regularly playing virtual/mobile/computer/laptop games online or offline were asked to consume a capsule containing 300 mg of Stadice^®^ or placebo per day for seven days. Cognitive ability tests, that were part of the NIMHANS Neuropsychological Battery and Auditory verbal learning tests were used to assess the cognitive health effects. Psychological stress response, anxiety, mood, subjective working memory, and cortisol levels were also assessed. All assessments were carried out at the baseline and at the end of the study.

Results

Stadice^®^ was found to significantly improve mental speed, attention, working memory, response inhibition, and verbal learning and memory as evidenced by the results of the multiple cognitive ability tests. Additionally, Stadice*^®^* showed beneficial responses in managing psychological stress in terms of handling nervousness, irritability, or mood swings. No safety concerns were found in the laboratory safety test parameters as they were within the normal physiological range and no adverse events were reported in this study.

Conclusion

The proprietary *Mangifera indica* extract (Stadice^®^) holds promise for enhancing cognitive abilities in healthy adults, particularly those engaged in esports. Improvements in learning, memory, mental speed, attention, response inhibition, and working memory among participants supplemented with Stadice^®^ were observed with a good safety profile. Further exploration is warranted to ascertain its broader applicability as well as to elucidate the possible mechanisms.

## Introduction

In recent years, the global landscape of sports and entertainment has been dramatically transformed by the rise of electronic sports, commonly known as esports. Esports refers to organized competitive gaming, where professional players and teams compete against each other in various video games, often in front of large audiences, both online and offline [1]. The esports industry has experienced exponential growth, fueled by advancements in technology, widespread internet access, and the increasing popularity of video games across all demographics. What was once a niche subculture has now evolved into a multi-billion-dollar industry [2], captivating millions of fans worldwide.

Cognition refers to mental action and encompasses various aspects of intellectual function including basic functions like attention, learning, memory, and working memory and complex functions like decision-making. Esports is a mentally challenging endeavor as it involves a significant cognitive demand, requiring players to engage in complex decision-making, strategic planning, rapid information processing, spatial awareness, hand-eye coordination, and adaptability. Esports games often involve split-second decisions that can have a significant impact on the outcome of a match. Players must constantly assess the situation, anticipate their opponents' moves, and choose the most effective strategies to achieve their objectives. Esports games are fast-paced and dynamic, requiring players to process vast amounts of information quickly and accurately. Players must stay adaptable, learning new techniques, adjusting their playstyles, and innovating to stay competitive in the ever-changing landscape of esports [3]. Excessive involvement in esports could result in overexertion, burnout, and a lack of diverse activities, all of which can negatively impact cognitive function, attention span, and emotional well-being.

Extended periods of watching television and using computers and mobile phones can also significantly impact cognitive health. Prolonged exposure to screens can lead to digital eye strain, characterized by symptoms such as headaches, eye discomfort, and difficulty focusing. Moreover, excessive screen time can disrupt sleep patterns, affecting cognitive function and mood regulation, which in turn could lead to stress and accumulation of reactive oxygen species [2,4]. Clinically studied supplements when used along with a healthy diet, adequate sleep, and regular exercise can potentially support esports athletes by enhancing cognitive function, physical performance, and overall well-being. For example, caffeine has been extensively studied and is known to improve alertness, focus, and physical performance in a variety of contexts, including esports [5,6]. *Mangifera indica*, commonly known as mango, has been used in traditional systems of medicine for various purposes, including culinary, nutritional, and medicinal applications. Mangiferin, of the xanthone group, is a major constituent of the leaves and stem bark of *Mangifera indica* L. (Anacardiaceae) and has antioxidant [7-9], immunomodulatory [10] and anti-inflammatory activities [7-9]. Mango extracts were reported to be safe at a preclinical level in acute oral [11-13], 28-day [12], and 90-day [14] repeated dose toxicity studies. Mangiferin was also reported to be non-genotoxic and non-clastogenic [15]. Stadice®, a proprietary standardized extract of *Mangifera indica* was found to be safe with LD_50_ of >5000 mg/kg bw in rats in an acute oral toxicity study (unpublished). Past clinical studies on mango leaf extract reported its cognitive health benefits and safety after a single dose of 500 mg or 300 mg of the standardized mango leaf extract [16,17]. This study is the first to assess both the cognitive health benefits and the safety of a standardized mango leaf extract given at a dose of 300 mg per day for seven days. Here, we evaluated the clinical efficacy and safety of Stadice®. The primary objective of the study was to find the effect of Stadice® on specific cognitive domains, such as mental speed, focused attention, sustained attention, working memory, and response inhibition, as well as on verbal learning and memory and visuo-spatial construction. The secondary objective of the study was to evaluate the effect of Stadice® on stress, mood changes, subjective working memory, cortisol level, and safety.

## Materials and methods

Trial design

A randomized, double-blind, placebo-controlled study was designed to evaluate the efficacy and safety of Stadice® for enhancement of cognitive abilities in healthy who were regularly playing virtual/mobile/computer/laptop games online or offline. The study was initiated after approval from the Pranav Diabetes Center Ethics Committee, informed consent from participants was obtained, and it was registered in the Clinical Trials Registry - India (CTRI/2023/02/050063). This study was conducted at a Maayra Emotional Wellness Centre, Bengaluru, India in compliance with the approved protocol, requirements of the Indian Council of Medical Research (ICMR) ethical guidelines, the International Council for Harmonization of Technical Requirements for Pharmaceuticals for Human Use (ICH) E6(R2) harmonized Guidance for Good Clinical Practice (GCP) (step 5), the New Drugs and Clinical Trials (NDCT) Rule 2019 and Declaration of Helsinki. A total of 60 healthy adults were recruited using opportunity sampling based on specified inclusion and exclusion criteria and were randomized into two groups in the ratio of 1:1 with 60 subjects each in Stadice® and placebo groups. The randomization process was carried out using a computer-generated random sequence. This sequence allocated each participant to one of the two study groups in a 1:1 ratio. This method ensured that assignment to either group was unbiased and independent of any external factors, thereby promoting an equitable distribution of participants across both groups. The double-blind study design ensured that both participants and investigators were unaware of the intervention assignments throughout the study duration. The blinding process in this study was further fortified by the incorporation of unique subject identification numbers (ID) and randomization codes. Each participant was assigned a distinct identification number, ensuring confidentiality and privacy throughout the study. The randomization codes, generated by an independent biostatistician, were then linked to the subject ID. Stadice® and placebo group subjects received their respective capsule products to be taken at a dose of 300 mg per day for seven days. Cognitive assessments were carried out on day 1 (baseline) as well as on day 7 after the last dose.

Participants

Healthy males and females aged between 18 and 45 years and who were willing to give voluntary written informed consent for participation in the study and willing to comply as per the protocol requirements were screened for inclusion in the study. Subjects were considered for inclusion in the study if they were in good health and were regularly (minimum three hours per week) playing virtual/mobile/computer/laptop games online or offline. Subjects with high blood pressure (systolic over 159 mmHg or diastolic over 99 mmHg), body mass index outside the range of 18.5 to 35 kg/m^2^, women who were pregnant, actively seeking to become pregnant, or lactating, learning and/or behavioral difficulties such as dyslexia or attention-deficit/hyperactivity disorder (ADHD), visual impairment that could not be corrected with glasses or contact lenses (including color-blindness), smoked tobacco or vaped nicotine or used nicotine replacement products more than two times per day, exceeded excessive caffeine intake (>500 mg per day) or had taken antibiotics or dietary supplements (e.g., vitamins, omega 3 fish oils, etc.) in the last four weeks, currently participating in other clinical or nutrition intervention studies or had participated in the past four weeks, undergoing treatment for alcohol or drug abuse in the last one month, undergoing treatment for a psychiatric disorder in the last one month, suffered from frequent migraines that required medication (more than or equal to one per month), sleep disorders or were taking sleep aid medication, inability to speak with study personnel via Zoom or did not have a private area to perform the study activities were excluded from the study.

Interventions and treatment compliance

Stadice® and placebo capsules used in the study were supplied by Zeus Hygia Lifesciences Pvt Ltd, Hyderabad, India. Placebo capsules used in the study looked like that of test capsules in terms of color, size, and shape. Each test capsule contained 300 mg of Stadice® powder. Stadice® is standardized to mangiferin and the ingredient is processed using aqueous-based insolib technology. The insolib technology has been developed to improve the solubility and stability of herbal actives, which tend to be unstable in lower pH environments. Each capsule of placebo contained 300 mg of microcrystalline cellulose. All subjects were asked to take one capsule once a day orally in the morning after food for seven days. Every effort was made to encourage subjects to comply with the dosage as per the protocol during the completed study. Subjects were instructed to bring or return their unused study product to the investigator at the end of the study visit. The assessment of treatment compliance involved recording relevant information on the dispensed, consumed, and returned study products in the case report form (CRF). A subject diary was also dispensed to record the consumption of test products daily during the study duration.

Outcomes

The primary outcome measures of this study included changes in mental speed, focused attention, sustained attention, working memory, response inhibition, verbal learning and memory, and visuospatial construction. Changes in the psychological stress response, mood changes, subjective working memory, plasma cortisol levels, safety, and tolerability were considered secondary outcome measures. All the tests were carried out by a clinical psychologist at baseline on day 1 and after day 7. Tests used in the assessment of mental speed, focused attention, sustained attention, working memory, response inhibition, verbal learning and memory, and visuospatial construction were part of the NIMHANS Neuropsychology Battery and were carried out as per Sistla et al. [18]. The NIMHANS Neuropsychological Battery scale developed by the National Institute of Mental Health and Neurosciences (NIMHANS) in India, typically consists of a series of tasks and tests that are administered and scored according to standardized procedures to provide a comprehensive evaluation of cognitive abilities. Digit Symbol Substitution Test (DSST), Color Trails Test 1 and 2, Digit Vigilance test, Verbal N Back Test, Stroop Test, Auditory Verbal Learning Test, and Complex Figure Test were used for assessing mental speed [19,20], focused attention [21], sustained attention [22], working memory [23], response inhibition [20], verbal learning and memory [24], and visuospatial construction [25] respectively.

The DSST is described as a pencil and paper-test of psychomotor performance. In this test, the subject is provided with a key grid containing numbers and matching symbols, along with a test section that includes numbers and empty boxes. The task is to fill as many empty boxes as possible with a symbol that corresponds to each number. The time taken for completion of the test indicates the scores. The shorter the time better the score. The DSST is known for its high test-retest reliability [26]. The Color Trails Test is a neuropsychological test that measures a person’s ability to maintain focused attention and task switching. It consists of two parts. Color Trails Test 1: in this part, the individual is asked to connect circles numbered 1 through 25 in ascending order. The circles are colored pink and yellow, but the color is not relevant to the task. Color Trails Test 2: this part is like the first, but this time the individual is asked to connect circles in ascending order, alternating between pink and yellow colors. The time taken to complete each part is recorded, with a shorter completion time indicating better cognitive function. This test is particularly useful in assessing cognitive impairment and damage to the frontal lobes, which are responsible for executive functions such as attention and task switching. It is also less influenced by language and cultural background compared to similar tests, making it a valuable tool in diverse populations. The Digit Vigilance Test assessed sustained attention by measuring the subject's ability to perform a task within a brief timeframe. Performance on the test was determined by the time taken, with longer durations indicating poorer performance. The error score was calculated as the sum of the number of omissions. To assess the change in working memory, the Verbal N-back Test 1 and 2 were employed, measuring both hits and errors. It mainly refers to the capacity to hold and manipulate information in an ongoing process. The score in each test was determined by the number of correct responses denoted by the number of hits, with higher scores indicating better performance. Response inhibition, assessed by the Stroop Test, measured the ease with which a perceptual set can be shifted both to conjoin demands and suppress a habitual response in favor of an unusual one. The duration needed to read printed words and to identify the color of printed words was converted into seconds. The time taken to read the printed words was subtracted from the time taken to read the color to get the Stroop effect score. The higher the score, the poorer the performance. The Auditory Verbal Learning Test (AVLT) measured the changes in verbal learning and memory assessed. The total number of words recalled across all five trials constituted the AVLT-total score. Memory performance was determined by the number of words correctly recalled in the immediate recall trial and delayed recall trial. The number of hits or correct responses was scored separately. A higher score indicated better performance. Complex Figure Test assessed the patient’s ability to reproduce complex geometric shapes. The correctness of reproduction was assessed according to the scoring system given in the test manual, with higher the score, the better the performance.

Psychological stress response was assessed using a questionnaire that contained five questions pertaining to their perceived stress, symptoms of stress, their feeling, their strategies to cope with stress, and their experience of worries. Subjects must provide a score for each question and the scores range from 1 to 5 with 1 indicating “not at all” and 5 indicating “most of the times.” The lower the score, the better the stress level of the subjects. Mood changes were assessed using the State-Trait Anxiety Inventory (STAI) and Mood Measure Questionnaire. STAI is a widely used self-report measure to assess the intensity of feelings of anxiety. The inventory consists of two scales, one for state anxiety (STAI-S) and one for trait anxiety (STAI-T), each containing 20 items. Respondents rate their feelings on a scale [27]. The mood measure questionnaire contained five questions and subjects must provide a score for each question; the scores range from 1 to 10 with 1 indicating “very low” and 10 indicating “very high.” The higher the score better the mood of the subjects. Subjective working memory was assessed using a questionnaire consisting of 15 questions. Subjects must provide a score for each question ranging from 1 indicating “strongly disagree” to 5 indicating “strongly agree.” Plasma cortisol levels were measured in 50% of the study population in each group by electrochemiluminescence immunoassay method using a cortisol kit (Elecsys® Cortisol II, Roche Diagnostics, Indianapolis, USA). The investigator was responsible for monitoring the safety of the study subjects who had entered the study and alerting the designee of the sponsor to any event that seemed unusual, even if an event could be considered an unanticipated benefit to the subject. Any adverse effect that occurred during the study period was evaluated for start date, end date, severity, relation to the study, outcome, concomitant medicine, and end date of the adverse event causality. Thus, the safety of Stadice® was assessed during the conduct of the study.

Statistical methods

A sample size of 60 completed subjects was considered adequate for this study, but no formal sample size calculation was carried out. The efficacy analysis population comprised all subjects who successfully completed the study without any major protocol deviations. Quantitative data obtained at different time points (t) underwent tests of normality, namely the Shapiro-Wilk test. Depending on the distribution nature, appropriate descriptive and inferential statistics were selected. Normally distributed data was characterized using mean and standard deviation, while non-normally distributed data was presented using median and interquartile range. Qualitative data was expressed in terms of frequency and percentage. A line diagram was employed for graphical representation, illustrating the trend in the outcome variable over time. The null hypothesis was generally rejected when a p-value less than or equal to 0.05 (5% significance level) was revealed by statistical procedures utilizing IBM SPSS Statistics for Windows, Version 19 (Released 2010; IBM Corp., Armonk, New York, USA).

## Results

Baseline demographics and participant flow

**Figure 1:**
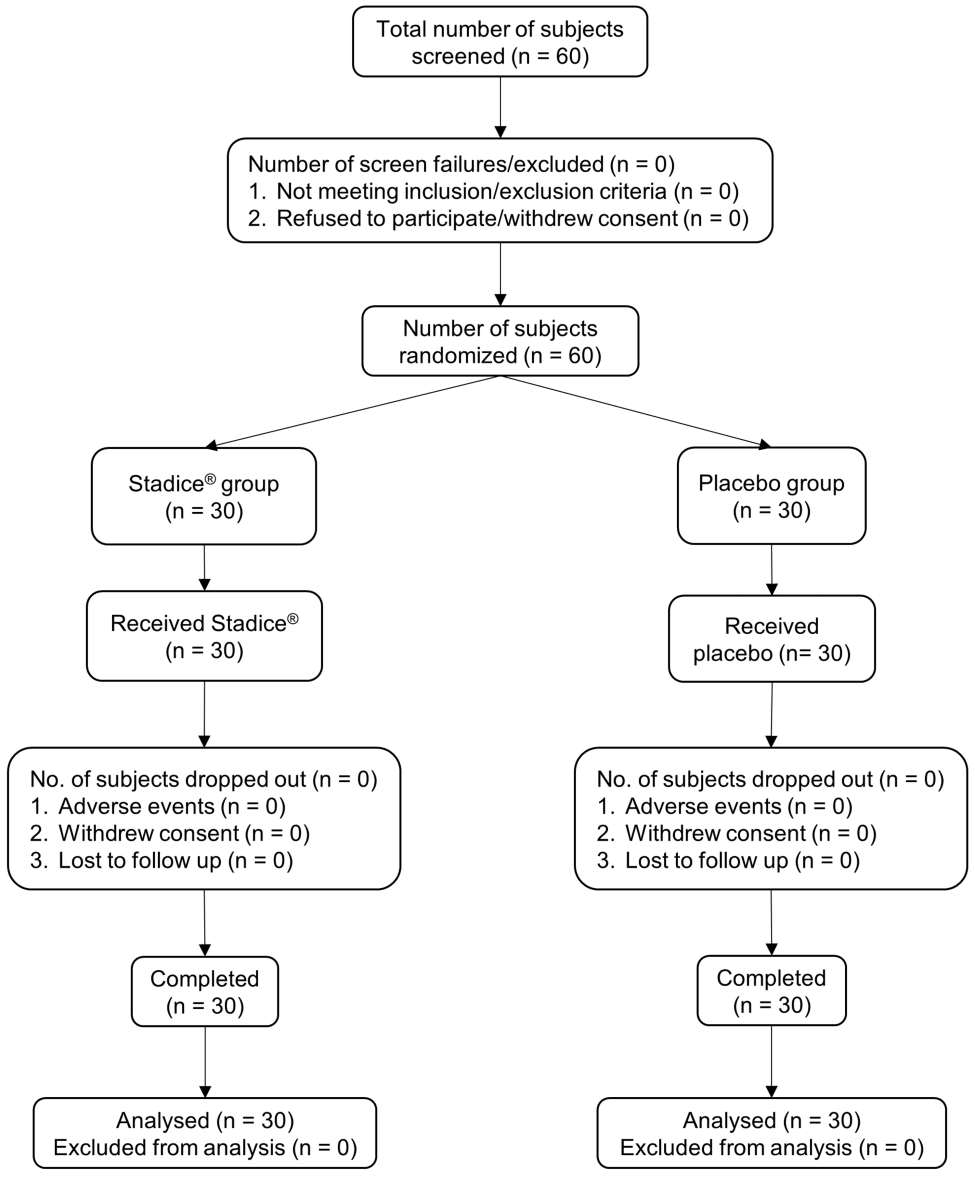
Flow of participants in the study

Recruitment was carried out by advertisements through CRO social media handles and through professional recruitment services. The study was conducted for a duration of about 60 days between September 2023 and October 2023. A total of 60 healthy subjects enrolled in the study as per the inclusion and exclusion criteria. All the enrolled subjects were randomly assigned to the test group and placebo group in the ratio of 1:1 with 30 subjects in the test group and 30 subjects in the placebo group. All the subjects were males with a mean age of 35.06 ± 5.76 years and 35.73 ± 5.71 years in the test group and placebo group, respectively. The baseline characteristics of the study population are shown in Table 1. The flow of participants in the study is shown in Figure 1. There were no dropouts in the study and all the subjects completed the study without any deviation from the study protocol. It was observed that subjects achieved a treatment compliance rate of 90%, indicating a high level of adherence to the study interventions. Therefore, data from all the subjects were included in the analysis.

**Table 1:** Baseline characteristics of the study population Each value represents mean ± SD of n = 30

Study parameters	Stadice^®^ group (n = 30)	Placebo group (n = 30)	
Age (yrs)	35.06 ± 5.76	35.73 ± 5.71	
Height (cm)	164.33 ± 5.53	163.73 ± 4.57	
Weight (kg)	60.24 ± 4.73	60.32 ± 4.33	

Outcomes and estimations

Cognitive Ability Tests

**Figure 2:**
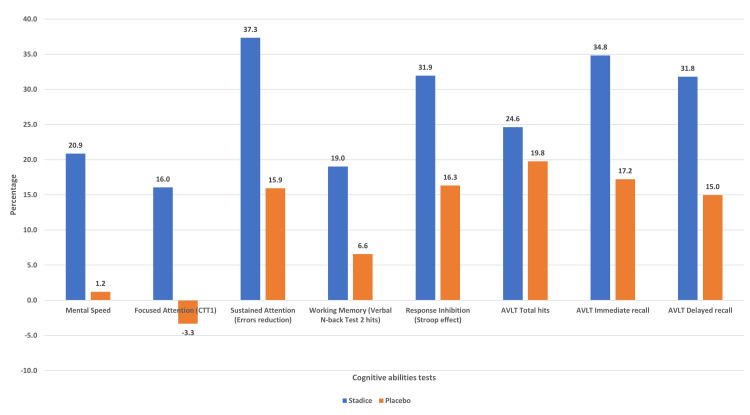
Percentage improvements in cognitive ability tests AVLT: Auditory Verbal Learning Test; CTT1: Color Trails Test 1

Results obtained from the NIMHANS Neuropsychology Battery and AVLT tests are presented as mean ± SD in Table 2 and the results of some cognitive ability tests in terms of percentage improvement are graphically presented in Figure 2. Stadice® group showed statistically significant improvement compared to placebo in mental speed (20.9% vs. 1.2%, p < 0.05) as assessed by DSST, focused attention (16.0% vs. -3.3%, p < 0.05) as assessed by Color Trails Test 1, sustained attention (37.3% vs. 15.9%, p < 0.05) as assessed by errors reduction in digit vigilance test, response inhibition (31.9% vs. 16.3%, p < 0.05) as assessed by Stroop Test, AVLT immediate recall (34.8% vs. 17.2%, p < 0.05) and AVLT delayed recall (31.8% vs. 15.0%, p < 0.05) as assessed using AVLT. Stadice® group exhibited statistically significant (p < 0.05) improvement (19.0%) from the baseline, in the case of working memory as assessed using Verbal N-back test 2 - hits compared to the placebo group, which showed a minimal change of 6.6% improvement. In the AVLT, a significant improvement from the baseline was observed in AVLT trial 1 (47.3%, p < 0.001), trial 2 (35.4%, p < 0.005), trial 3 (17.9, p < 0.05), trial 4 (18.5% p < 0.05) and total hits (24.6% p < 0.01) in the test group but significant improvement from the baseline was observed in the placebo group only for AVLT trial 1 (42.0%, p < 0.001), trial 2 (32.6%, p < 0.005), and total hits (19.8%, p < 0.05). However, the differences observed between the groups in the Verbal N-back test 2 - hits and AVLT tests were not significant. No statistically significant differences were observed in other cognitive tests in both within the group analysis and between the groups analysis.

**Table 2:** Effect of Stadice® on cognitive ability tests Each value is represented as mean ± SD of n = 30 *: p < 0.05; ^**^: p < 0.01; ^***^: p < 0.005; ^****^: p < 0.001 significantly different from respective baseline; ^#^: p < 0.05 significantly different between the groups; ^a^: hits - number of correct answers; ^b^: errors - number of wrong answers

Cognitive abilities tests	Stadice® group (n = 30)		Placebo group (n = 30)		p-value between the groups
	Baseline	Day 7	Baseline	Day 7	
Digit Symbol Substitution Test (sec)	259.13 ± 58.10	205.07 ± 38.66^**^	253.34 ± 88.24	250.38 ± 56.03	p < 0.05
Color Trails Test 1 (sec)	81.20 ± 28.33	68.17 ± 20.53^*^	71.48 ± 24.29	73.86 ± 34.51	p < 0.05
Color Trails Test 2 (sec)	145.47 ± 36.44	132.37 ± 48.63	127.72 ± 35.39^#^	110.24 ± 32.95	p > 0.05
Digit vigilance test (sec)	575.03 ± 114.05	515.30 ± 97.89	548.52 ± 132.10	489.14 ± 111.80	p > 0.05
Digit vigilance test - errors^a^	11.33 ± 17.91	7.10 ± 6.19^***^	9.48 ± 8.42	7.97 ± 8.50^*^	p < 0.05
Verbal N-back Test 1 - hits^a^	7.97 ± 1.81	8.53 ± 0.68	8.14 ± 1.66	8.41 ± 1.18	p > 0.05
Verbal N-back Test 1 - errors^b^	2.63 ± 3.00	1.47 ± 1.70	2.03 ± 2.78	1.62 ± 1.97	p > 0.05
Verbal N-back Test 2 - hits^a^	5.57 ± 1.76	6.63 ± 1.59^*^	5.79 ± 2.27	6.17 ± 1.71	p > 0.05
Verbal N-back Test 2 - errors^b^	5.83 ± 2.97	4.07 ± 2.1	5.38 ± 2.86	4.66 ± 2.44	p > 0.05
Stroop test (sec)	148.90 ± 75.70	101.33 ± 34.03^***^	166.38 ± 61.10	139.24 ± 79.77^*^	p < 0.05
Auditory verbal learning test score, Trial 1 - hits^a^	6.40 ± 1.77	9.43 ± 1.92^****^	6.10 ± 1.88	8.66 ± 2.58^****^	p > 0.05
Auditory verbal learning test score, Trial 2 - hits^a^	8.07 ± 1.96	10.93 ± 2.39^***^	7.72 ± 2.52	10.24 ± 2.26^***^	p > 0.05
Auditory verbal learning test score, Trial 3 - hits^a^	9.90 ± 1.83	11.67 ± 1.99^*^	9.55 ± 2.64	10.93 ± 2.46	p > 0.05
Auditory verbal learning test score, Trial 4 - hits^a^	10.13 ± 2.26	12.00 ± 2.05^*^	9.97 ± 2.70	11.55 ± 2.61	p > 0.05
Auditory verbal learning test score, Trial 5 - hits^a^	10.40 ± 2.11	11.83 ± 2.10	10.83 ± 2.30	11.86 ± 2.84	p > 0.05
Auditory verbal learning test score, Total hits^a^	44.83 ± 7.32	55.87 ± 8.90^**^	44.17 ± 10.26	52.90 ± 10.98^*^	p > 0.05
Auditory verbal learning test score, List B No. Correct	4.80 ± 1.95	5.00 ± 1.60	5.24 ± 2.59	5.21 ± 2.14	p > 0.05
Auditory verbal learning test score, Immediate recall - hits^a^	8.73 ± 3.32	11.77 ± 2.03^***^	9.00 ± 3.00	10.55 ± 3.39^*^	p < 0.05
Auditory verbal learning test score, Delayed recall - hits^a^	8.93 ± 2.29	11.77 ± 2.40^***^	9.55 ± 2.84	10.98 ± 2.78	p < 0.05
Auditory verbal learning test score, hits^a^	13.40 ± 1.22	14.43 ± 0.77	13.69 ± 1.67	13.79 ± 2.65	p > 0.05
Complex figure test	28.48 ± 6.42	30.45 ± 4.43	28.97 ± 5.13	29.14 ± 4.21	p > 0.05

Effect of Stadice® on Psychological Stress

Results of the psychological stress response questionnaire (Table 3) indicated that the use of Stadice® could help subjects manage psychological stress better compared to placebo in terms of anxiety or nervousness and irritability or mood swings.

**Table 3:** Results of psychological stress response questionnaire Each value is represented as mean ± SD of n = 30 ^*^: p < 0.05; ^**^: p < 0.005, significantly different from the respective baseline. No significant difference between the groups.

S. No.	Psychological stress response questionnaire	Stadice^®^ group (n = 30)		Placebo group (n = 30)	
		Baseline	Day 7	Baseline	Day 7
1	Please rate your overall level of perceived stress in the past month	3.0 ± 2.0	3.0 ± 1.0	3.0 ± 0.0	3.0 ± 0.0
2	How often do you experience the following physical symptoms in response to stress?				
2.1.	Headache or migraines	1.0 ± 2.0	1.0 ± 1.0^*^	2.0 ± 2.0	1.0 ± 0.5^*^
2.2.	Muscle tension or pain	1.0 ± 1.0	1.0 ± 1.0	1.0 ± 2.0	1.0 ± 0.5^*^
2.3.	Digestive issues (e.g., Stomach-ache, indigestion)	1.0 ± 1.0	1.0 ± 0.25	1.0 ± 0.50	1.0 ± 0.5
2.4.	Fatigue or exhaustion	1.0 ± 1.0	1.0 ± 1.0	1.0 ± 1.0	1.0 ± 0.0
2.5.	Rapid heartbeat or palpitations	1.0 ± 0.0	1.0 ± 1.0	1.0 ± 0.0	1.0 ± 0.0^*^
3	Rate your feeling				
3.1.	Anxiety or nervousness	1.5 ± 2.0	1.0 ± 0.0^**^	1.0 ± 2.0	1.0 ± 1.0^*^
3.2.	Irritability or mood Swings	2.0 ± 1.25	1.0 ± 0.0^**^	1.0 ± 2.0	1.0 ± 1.0
3.3.	Feeling overwhelmed or unable to stop.	1.0 ± 2.0	1.0 ± 1.0	1.0 ± 1.5	1.0 ± 1.0
3.4.	Restlessness or difficulty relaxing	1.0 ± 1.25	1.0 ± 1.0	1.0 ± 2.0	1.0 ± 1.0
3.5.	Difficulty concentrating or making decisions.	1.5 ± 2.0	1.0 ± 1.0	1.0 ± 2.0	1.0 ± 1.0*
4	How often do you engage in the following coping strategies when faced with stress?				
4.1.	Engaging in physical exercise or activity	3.0 ± 2.0	2.0 ± 2.0^*^	3.0 ± 3.0	2.0 ± 2.0
4.2.	Seeking social support from friends or family	2.0 ± 2.0	2.0 ± 2.0	3.0 ± 3.0	2.0 ± 1.0
4.3.	Engaging in relaxation techniques (e.g., deep breathing. meditation)	2.0 ± 2.0	2.0 ± 2.0	3.0 ± 2.0	2.0 ± 2.0
4.4.	Taking breaks or engaging in enjoyable activities	3.0 ± 2.25	3.0 ± 1.0	3.0 ± 1.0	3.0 ± 1.0
4.5.	Seeking professional help or counseling	1.0 ± 0.25	1.0 ± 0.25	1.0 ± 1.5	1.0 ± 1.0
5.	How frequently do you experience intrusive thoughts or worries related to stressful situations?	2.0 ± 1.0	2.0 ± 0.0	2.0 ± 0.0	2.0 ± 1.0

Effect of Stadice® on Anxiety

The mean STAI score in the Stadice® group was 76.87 ± 13.69 (n = 30) during baseline evaluation and was dropped to 65.97 ± 14.20 (n = 30) by the end of the study, which was statistically significant (p < 0.005) compared to the baseline. However, the mean STAI score in the placebo group also showed a similar drop from 74.79 ± 15.81 (n = 30) at baseline to 68.17 ± 12.05 (n = 30) by the end of the study, which was statistically significant (p < 0.005) compared to the baseline.

Effect of Stadice® on Mood

The below-presented mood measure questionnaire table (Table 4) reflects that neither of the groups showed any promising improvement in the state of mood for the studied subjects.

**Table 4:** Results of the mood measure questionnaire Each value is represented as mean ± SD of n = 30 ^*^: p < 0.05, ^**^: p < 0.01, ^***^: p < 0.005, significantly different from the respective baseline. No significant difference between the groups

S. No.	Mood measure questionnaire	Stadice^® ^group (n = 30)		Placebo group (n = 30)	
		Day 0	Day 7	Day 0	Day 7
1	How would you rate your overall mood on an average day?	7.0 ± 2.25	8.0 ± 2.25	7.0 ± 2.50	8.0 ± 1.5^***^
2	How frequently do you engage in activities or practices specifically aimed at improving your overall mood on an average day?	7.0 ± 4.0	8.0 ± 2.0	7.0 ± 3.0	8.0 ± 4.0^*^
3	How satisfied are you with your current methods of mood enhancement? Are there any areas where you feel improvement is needed?	6.5 ± 3.0	7.5 ± 3.0^*^	7.0 ± 3.0	7.0 ± 3.0
4	In your opinion, how important is mood enhancement for overall mental health and quality of life?	8.5 ± 3.0	9.0 ± 2.0	8.0 ± 2.0	9.0 ± 2.0^**^
5	How would you rate your mood immediately after engaging in the study?	7.0 ± 2.0	9.0 ± 2.0	8.0 ± 2.0	7.0 ± 2.5

Effect of Stadice® on Working Memory

Results (Table 5) tabled below of the subjective working memory questionnaire did not show any significant effect both in the placebo group and in the Stadice® group.

**Table 5:** Results of the working memory questionnaire Each value is represented as mean ± SD of n = 30 ^*^: p < 0.05 significantly different from respective baseline; ^#^: p < 0.05 significantly different between the groups

S. No.	Working memory questionnaire	Stadice^® ^(n = 30)		Placebo (n = 30)	
		Day 0	Day 7	Day 0	Day 7
1	I can hold and manipulate multiple pieces of information in my mind simultaneously.	4.0 ± 1.0	4.0 ± 1.0	4.0 ± 1.0	4.0 ± 1.0
2	I find it easy to remember and follow multi-step instructions.	4.0 ± 1.0	4.0 ± 0.0^*^	4.0 ± 1.0	4.0 ± 0.0
3	I can mentally juggle multiple tasks or priorities without getting overwhelmed.	4.0 ± 1.0	4.0 ± 1.0	4.0 ± 1.0	4.0 ± 0.5^*^
4	I have a good memory for phone numbers. addresses. or other numerical information.	4.0 ± 1.0	4.0 ± 1.0	5.0 ± 1.0	5.0 ± 1.0
5	I can quickly recall important facts or details from recent conversations.	4.0 ± 0.0	4.0 ± 0.0	4.0 ± 1.0	4.0 ± 1.5
6	I can easily switch my attention between different tasks or topics.	4.0 ± 1.0	4.0 ± 1.0	4.0 ± 0.5	4.0 ± 0.5
7	I often find myself mentally rehearsing or repeating information to remember it.	4.0 ± 1.0	4.0 ± 1.0	4.0 ± 1.0	4.0 ± 1.0
8	I have a strong ability to concentrate and stay focused on a task.	4.0 ± 0.50	4.0 ± 0.0	4.0 ± 1.0	4.0 ± 0.5
9	I can mentally visualize and manipulate objects or spatial relationships.	3.0 ± 1.0	3.0 ± 1.0^#^	4.0 ± 1.0	4.0 ± 0.0^*^
10	I can remember and use relevant information from past experiences to solve problems.	4.0 ± 1.0	4.0 ± 0.0	4.0 ± 0.0	4.0 ± 0.5
11	I tend to forget important details or instructions if I don't write them down.	3.0 ± 2.0	3.0 ± 2.0	3.0 ± 2.0	3.0 ± 1.0
12	I often have to make an effort to stay organized and keep track of my task.	4.0 ± 1.0	4.0 ± 1.0	4.0 ± 1.0	4.0 ± 0.0
13	I can mentally break down complex problems into smaller. manageable parts.	4.0 ± 0.0	4.0 ± 1.0	4.0 ± 0.5	4.0 ± 1.0
14	I am good at remembering people's names and connecting them to faces.	5.0 ± 1.0	5.0 ± 1.0	4.0 ± 1.0	5.0 ± 1.0
15	I find it challenging to keep track of multiple conversations happening simultaneously.	3.0 ± 1.0	4.0 ± 1.0	4.0 ± 1.0	4.0 ± 1.0

Effect of Stadice® on Cortisol

Subjects in the Stadice® group and placebo group did not show any statistically significant changes in the levels of cortisol at the end of the study compared to the baseline and between the groups. The levels of cortisol seen in both groups were within the normal physiological levels.

Safety of Stadice®

It was observed that subjects achieved a treatment compliance rate of 90%, indicating a high level of adherence to the study intervention. No clinically significant changes were observed in the vital signs viz., temperature, pulse rate, systolic blood pressure, diastolic blood pressure, and respiratory rate and the values remained within the normal range. Also, there were no clinically significant changes seen in the results of the hematology (Table 6), biochemistry, liver function tests, and renal function tests.

**Table 6:** Results of the hematology parameters *: p < 0.05 compared to the baseline of the placebo group

Hematology parameters	Baseline		Day 7	
	Stadice^®^ (n = 30)	Placebo (n = 30)	Stadice^®^ (n = 30)	Placebo (n = 30)
Hemoglobin (g/dL)	13.28 ± 1.09	14.33 ± 1.87	13.24 ± 1.11	14.26 ± 1.96
Packed cell volume	41.29 ± 2.98	42.83 ± 3.89	41.61 ± 3.2	43.68 ± 5.03
RBC count (×10^6^ cells/mm^3^)	5.34 ± 0.85	5.11 ± 0.57	5.38 ± 0.86	5.19 ± 0.47
Total leucocyte count (cells/mm^3^)	7111.11 ± 1616.06	11391.67 ± 1655.46	7150 ± 1502.64	6625 ± 1820.65
Neutrophils (%)	54.82 ± 7.07	57.83 ± 7.71	53.66 ± 7.83	54.22 ± 9.95
Lymphocytes (%)	31.97 ± 7.37	31.73 ± 6.86	33.31 ± 6.93	33.68 ± 8.53
Eosinophils (%)	4.52 ± 2.04	3.32 ± 1.28	4.52 ± 2.3	3.61 ± 1.32
Monocytes (%)	7.93 ± 1.57*	6.46 ± 1.97	7.69 ± 1.52	7.74 ± 1.22
Basophil (%)	0.76 ± 0.35	0.67 ± 0.18	0.82 ± 0.34	0.76 ± 0.23
Platelet count (×10^3^ cells/mm^3^)	318.89 ± 98.38	287.92 ± 60.73	329.78 ± 95.37	292.5 ± 64.48

## Discussion

*Mangifera indica*, also known as the mango, has been a valued herb in Ayurvedic and indigenous medical systems for over 4,000 years. One of the most popular tropical fruits, the mango, possesses strong antioxidant, anti-lipid peroxidation, immunomodulating, cardiotonic, hypotensive, wound healing, antidegenerative, and antidiabetic activities due to its presence of polyphenolic antioxidants and glucosyl xanthones [10]. Chronic accumulation of lipopolysaccharide (LPS) can induce neuroinflammation, ultimately leading to cognitive deficits. Heme oxygenase 1, a key enzyme in antioxidant and anti-inflammatory pathways, is downregulated during this process. Mangiferin supplementation was found to decrease LPS-induced IL-6 production and upregulate heme oxygenase-1 expression in the hippocampus, suggesting its potential as a neuroprotective agent [7]. Mangiferin, an antioxidant and anti-inflammatory agent, was shown to prevent sleep deprivation (SD)-induced behavioral and neurochemical changes in mice. Sleep deprivation in mice significantly impaired learning and cognition, as evidenced by tests like the Morris water maze and novel object recognition tasks. Brain-derived neurotrophic factor (BDNF) plays an important role in neuronal growth and survival, synaptic plasticity, synaptogenesis, long-term potentiation (LTP), and memory function. Levels of BDNF were found to decrease in subjects with age-related cognitive decline and Alzheimer’s disease. Pretreatment with mangiferin provided protection against these SD-induced neurobehavioral and neurochemical changes. Additionally, mangiferin offered beneficial effects against the increase in pro-inflammatory cytokine levels in the periphery and brain, reduced oxidative stress, and restored the decreased levels of BDNF in both plasma and hippocampus [8]. Recent studies investigating brain-relevant enzyme inhibition have shown that mangiferin significantly inhibited catechol-O-methyltransferase (COMT), the enzyme responsible for degrading catecholamine neurotransmitters. COMT's catabolic pathway is particularly prevalent in brain tissue with low levels of catecholamine reuptake transporters. COMT inhibition primarily affects dopaminergic function in the prefrontal cortex and hippocampus, potentially leading to improvements in working memory, selective attention, and executive function [16]. LTP can be conceptualized as the persistent strengthening of synaptic connections and is a major cellular mechanism behind learning and memory. Mangiferin and *Mangifera indica* extract were reported to increase LTP [16]. Mangiferin ameliorated the scopolamine-induced learning deficits in mice, which was thought to be partly due to its inhibitory mechanism against acetylcholinesterase [28]. Mangiferin was reported to exhibit its neuroprotective effects by multiple mechanisms including its antioxidant, acetylcholinesterase inhibition, and lipoxygenase inhibition [9].

Multiple neuropharmacological mechanisms reported on mangiferin and *Mangifera indica* prompted us to investigate the cognitive health-promoting properties of Stadice® in this randomized, double-blind, placebo-controlled clinical study in subjects who were actively engaged in esports. Results obtained in the cognitive abilities tests conducted using NIMHANS Neuropsychology Battery and auditory verbal learning tests showcase the effect of Stadice® on mental speed, attention, working memory, and verbal learning and memory. Additionally, Stadice® seems to have the potential to manage psychological stress responses in terms of handling nervousness, irritability, or mood swings. A single oral dose of 300 mg of mango leaf extract standardized to mangiferin has been reported to have improved performance accuracy across the tasks in the battery and showed improvement across all three tasks viz., rapid visual information processing, serial 3s, and serial 7s subtraction tasks without any negative effects on mood [17]. In another report, a single oral dose of 500 mg of mango leaf extract was found to show significant spectral changes in brain electrical activity in cortical regions like that of LTP during cognitive challenges, tend to show faster reaction time and improved all the scores for Profile of Mood States [16]. The cognitive health benefits observed in our study also were without any side effects as there were no reports of any adverse events during the study and the results of the safety evaluation parameters were all within the normal physiological range. We did not get significant effects in the measures of anxiety assessed using the State-Trait Anxiety Inventory (STAI) and measures of mood assessed using the Mood Measure Questionnaire possibly because the subjects in the placebo seemed to have normal scores in the STAI and Mood Measure Questionnaire indicating the lack of any stress in the studied population that could impact these scores. Subjective working memory assessed using Working Memory Questionnaire did not show any significant difference possibly because of the lesser sample size for this subjective outcome measure. Also, we did not find any change in the cortisol level of subjects indicating the subjects involved in the study were not sufficiently stressed on account of their esports activities. Safety of the test substance Stadice®, used in this study was earlier tested for its safety in an acute oral toxicity study and Stadice® was found to be safe with LD_50 _>5000 mg/kg bw in Wistar rats (unpublished report). The safety of mangiferin and mango extract at preclinical levels has been previously reported and the mango extract and mangiferin were generally considered safe [11-14] and without any genotoxic effects [14,15,29]. Although the sample size was not calculated before the initiation of the study, a post hoc power analysis was conducted using G Power (version 3.1.9.7 for Windows, The G*Power Team, Germany) based on the results obtained for the primary outcome variable (NIMHANS Battery scale). The power of the study was found to be 80% with an effect size of 0.65 when the alpha was fixed at 5% indicating the sample size used in the study was adequate for this study. One of the limitations of this study could be the usage of questionnaires that were not validated for specific purposes in specific populations. Another limitation of the study is that while the video gaming duration was specified as a minimum of three hours per week, it was not subdivided into different hourly categories based on the subjects' playing time as the study is exploratory in nature. Nevertheless, this study is important in the sense that the test substance Stadice® used in this study is a unique formulation wherein the physicochemical properties of mangiferin in the formulation were enhanced using a patented insolib technology that minimizes the degradation of active substances in herbal extracts.

## Conclusions

The results from our clinical investigation suggest that Stadice® (*Mangifera indica* extract) exhibits considerable potential as an intervention for augmenting cognitive faculties in healthy adult populations, particularly those actively involved in e-sports activities. Our study indicates notable enhancements in learning and memory capabilities, alongside improvements in mental speed, attention, and working memory functions among participants. Importantly, our findings also indicate that Stadice® demonstrates a good safety profile. These promising results highlight Stadice® as a prospective tool in the realm of cognitive enhancement research, offering valuable insights into its efficacy and safety within specialized contexts such as e-sports. Further exploration and rigorous investigation are warranted to ascertain its broader applicability.
